# Intra‐set rest preserves barbell velocity during a free‐weight back squat exercise by shifting energy system contribution

**DOI:** 10.1113/EP093514

**Published:** 2026-03-19

**Authors:** Erik Hobein, Antonia Edel, Ivan Jukic, Alexander Ferrauti, Thimo Wiewelhove

**Affiliations:** ^1^ Department of Training and Exercise Science Faculty of Sport Science Ruhr University Bochum Bochum Germany; ^2^ Department of Cardiology and Angiology Contilia Group Essen Germany; ^3^ Department of Health, Sport and Wellbeing, Faculty of Social and Applied Sciences Abertay University Dundee UK; ^4^ Sport Performance Research Institute New Zealand (SPRINZ) University of Technology Auckland New Zealand; ^5^ Department of Fitness and Health IST University of Applied Sciences Düsseldorf Germany

**Keywords:** energy metabolism, oxygen consumption, exercise, physiology, cluster set, PCr‐La‐O2 method

## Abstract

In this study, we compared acute metabolic responses and barbell velocity loss (VL) during cluster set (CS) versus traditional set (TS) resistance exercise. Six strength‐trained individuals completed two randomized crossover sessions of back squats. The CS protocol included 30 s intra‐set rest intervals after the second and fourth repetitions, whereas the TS protocol used continuous repetitions. Barbell velocity, oxygen uptake and blood lactate concentrations were measured to derive model‐based indices of energy system engagement. CS were associated with better preservation of barbell velocity and lower blood lactate concentrations compared with TS. Model‐based indices suggested distinct metabolic patterns between set structures, with CS being associated with a higher alactic and lower lactic component than TS. Inter‐individual responses were consistent across most participants. These findings indicate that acute metabolic and mechanical responses differ between CS and TS configurations. CS were associated with reduced indices of metabolic stress alongside improved velocity preservation, highlighting that set structure can influence the acute physiological–performance profile of resistance exercise.

## Abstract (highlights)

- **What is the central question of this study?**
  Cluster sets might alter acute metabolic and mechanical responses. However, the patterns of energy system engagement during cluster sets remain unclear. This study examined whether cluster sets and traditional sets elicit different acute energy and performance profiles based on a model‐based approximation of energy system components.
- **What is the main finding and its importance?**
  Cluster sets were associated with a distinct metabolic profile alongside a better preservation of movement velocity. These findings suggest that different set structures elicit qualitatively different acute physiological responses, which might be relevant when selecting resistance exercise configurations.

## 1 INTRODUCTION

Resistance training is widely recognized as an important part of physical preparation that supports performance across various sports (Suchomel et al., 2016). Coaches use training strategies to target goals such as strength, hypertrophy and power, adjusting factors such as loading schemes, set configurations, rest intervals and autoregulated approaches (Hickmott et al., 2022; Suchomel et al., 2018). One popular autoregulation strategy, velocity‐based training, uses velocity loss (VL) thresholds to manage fatigue and optimize the training stimulus (Guppy et al., 2024; Weakley et al., 2021). However, reliance of velocity‐based training on specialized equipment makes it impractical for many, creating a need for alternative set structures that can limit VL intrinsically.

Cluster sets (CS) offer such an alternative (Jukic et al., 2022). Unlike traditional set (TS) structures, CS incorporate short intra‐set rest periods of 10–30 s (Haff et al., 2008). This rest is thought to allow partial phosphocreatine (PCr) resynthesis, helping to maintain higher movement velocities and power output (Jukic et al., 2020; Tufano et al., 2016). However, the relative contributions of anaerobic alactic, anaerobic lactic and aerobic pathways during CS remain unquantified (Tufano et al., 2016). Understanding these contributions is important, because they determine both the capacity to sustain movement velocity and the type of metabolic stress that drives specific training adaptations.

Therefore, the purpose of this study was to compare the acute effects of CS and TS on barbell velocity and energy pathway contributions during free‐weight back squats using the three‐component model (PCr‐La‐O_2_ method; phosphocreatine–lactate–oxygen method; Artioli et al., 2012; Beneke et al., 2002), adapted for intermittent exercise (Edel et al., 2024; Latzel et al., 2018). We hypothesized that CS would cause less VL than TS, primarily owing to a greater contribution from the anaerobic alactic energy system.

## 2 MATERIALS AND METHODS

### 2.1 Ethical approval

The study protocol was approved by the Ethics Committee of the Faculty of Sport Science, Ruhr University Bochum (approval number: EKS V 2024_18). All participants provided informed consent prior to participation. The study conformed to the *Declaration of Helsinki*, except for registration in a database.

### 2.2 Participants

Six strength‐trained individuals (one female: 24 years, 160 cm, 58.9 kg; and five males: 24 ± 5 years, 176 ± 3 cm, 72.4 ± 8.4 kg) participated in this study. Their relative strength in the back squat was 1.40 kg [kg body mass (BM)] ^−1^ for the female and 1.76 ± 0.21 kg (kg BM)^−1^ for the males. Inclusion criteria required participants to be 18–35 years old and to have ≥1 year of resistance training experience. Minimum one‐repetition maximum (1RM) standards were ≥1.00 kg (kg BM)^−1^ for females and ≥1.25 kg (kg BM)^−1^ for males. Participants abstained from other resistance training during the study.

Given the controlled, highly detailed crossover design and the exploratory nature of the study, a small sample size (*n* = 6) was considered adequate for exploratory within‐participant comparisons of acute metabolic and mechanical responses. This design allows each participant to serve as their own control, improving sensitivity to detect protocol‐dependent differences while minimizing between‐participant variability.

### 2.3 Study design

This study used a randomized, counterbalanced cross‐over design. Each participant completed three laboratory sessions separated by ≥24 h. The first session was for baseline measurements, including anthropometry and back squat 1RM testing to establish individual exercise loads. Participants were also familiarized with all procedures. In the subsequent two sessions, participants performed either the CS or TS exercise protocol in a randomized order.

### 2.4 Preliminary testing session

In the preliminary session, we collected anthropometric data and determined the back squat 1RM for each participant. To ensure consistency, all squats were performed with a self‐selected but controlled eccentric velocity and a maximal‐effort concentric phase. Squat depth was assessed individually by having participants squat as deeply as possible with a load of 50% BM while maintaining proper technique. The deepest position reached was recorded using a haptic barrier, which was then used to standardize squat depth across all subsequent sets and sessions. Barbell velocity was measured using a linear position transducer (Vitruve, SPEED4LIFTS S.L.) at 100 Hz, with mean propulsive velocity (MPV) used as the key metric. The 1RM was established using a standard progressive loading protocol, defined as the heaviest load lifted with correct technique.

### 2.5 Experimental sessions

After baseline blood lactate measurement and a standardized warm‐up, participants began the main exercise protocol: four sets of six back squats at 75% 1RM, with 3 min of rest between sets. The TS protocol involved six continuous repetitions. In contrast, the CS protocol incorporated a 30 s intra‐set rest after the second and fourth repetitions. Throughout the session, a portable spirometry system (MetaMax 3B‐R2, Cortex) continuously recorded respiratory data. A linear position transducer recorded barbell velocity for each repetition. Blood lactate was sampled 1 min before the final set and at 1, 3 and 5 min postexercise for analysis to capture peak lactate, which typically occurs shortly after exercise (Biosen C‐Line, EKF Diagnostics). During all intra‐set and inter‐set rest periods and for 15 min postexercise, participants remained seated and inactive.

### 2.6 Calculations and statistical analyses

**FIGURE 1:**
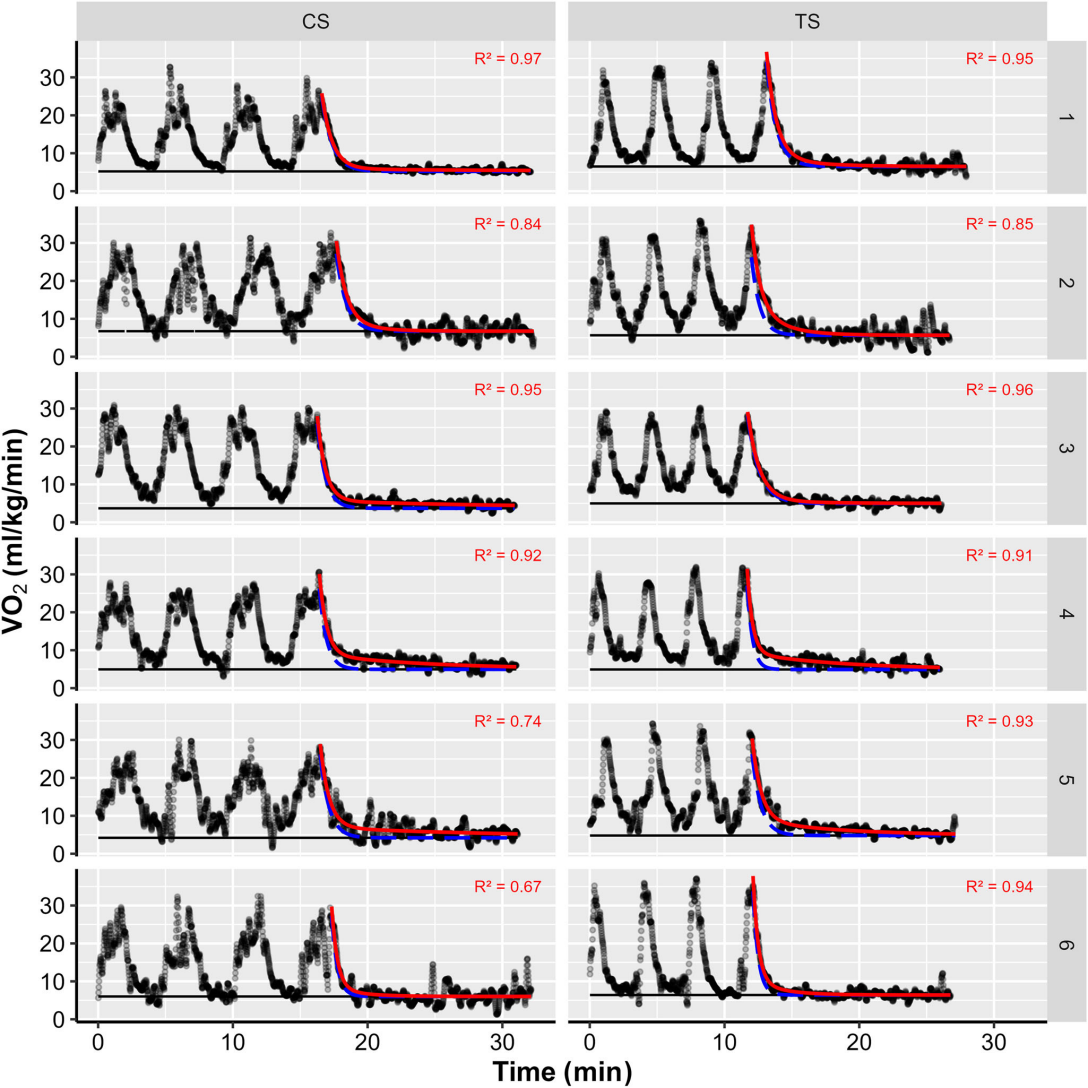
Visualization of the normalized oxygen uptake (${\dot{V}}_{O_{2}}$) during exercise with subsequent recovery for each participant (horizontal) and each training session (vertical) under cluster set (CS) and traditional set (TS) protocols (*n* = 6). The black horizontal line shows the baseline oxygen uptake, while the red continuous curve represents the calculated fit of the bi‐exponential function and the blue dashed curve the fast component.

Energy contributions of the aerobic, anaerobic alactic and anaerobic lactic systems were estimated using the PCr‐La‐O_2_ method, adapted for intermittent exercise (Artioli et al., 2012; Beneke et al., 2002; Edel et al., 2024; Latzel et al., 2018). Oxygen uptake (${\dot{V}}_{O_{2}}$) data from the portable spirometer were recorded as time‐series data and converted to seconds, smoothed using a centred 10‐point moving average, and transformed into litres per second. The start of the postexercise phase was defined manually and was modelled as:

$$\dot{V}O_{2}\left(t\right)=a\;\times \exp\left(-t/\tau_{a}\;\right)+b\;\times exp\left(-t/\tau_{b}\;\right)+c$$

where a and b are the amplitudes of the fast and slow components, $\tau_{a}$ and $\tau_{b}$ are the time constants, and c is oxygen uptake at baseline (see Figure 1). The parameters derived from this model were subsequently used for integration across all intra‐ and inter‐set rest periods, because shorter rest durations do not allow reliable curve fitting.

Aerobic energy was calculated by integrating baseline‐adjusted ${\dot{V}}_{O_{2}}$ over the full exercise phase, with corrections for intra‐set (30 s, CS only; fast component) and inter‐set (180 s, both protocols; fast and slow component) rest periods. Specifically, ${\dot{V}}_{O_{2}}$ integrals during all intra‐ and inter‐set rest periods were subtracted from the total ${\dot{V}}_{O_{2}}$ integral, such that the calculated energy reflected only the exercise periods. Anaerobic alactic energy was estimated by integrating the fast ${\dot{V}}_{O_{2}}$ component over the total recovery period, including the last 900 s rest, the three inter‐set rests and, for the CS protocol, the eight intra‐set pauses. Aerobic and anaerobic alactic oxygen consumption was converted to energy using the caloric equivalent of 20.92 kJ (L O_2_)^−1^ (Artioli et al., 2012). Anaerobic lactic energy was derived from the rise in blood lactate from baseline to peak, converted to O_2_ equivalents [3 mL O_2_ (kg BM)^−1^ mmol L^−1^] and subsequently to energy using the same caloric equivalent, in accordance with the PCr‐La‐O_2_ method (Artioli et al., 2012). These values are reported as model‐based indices to characterize relative contributions and should be interpreted qualitatively.

The VL was calculated as the percentage decrease in MPV (fastest vs. last repetition) for each set, averaged across the four sets in a session (Sánchez‐Medina & González‐Badillo, 2011).

Given the small sample size, both descriptive and exploratory inferential statistics were performed. Data processing and analysis were conducted in R (v.4.2.2; The R Foundation for Statistical Computing, Vienna, Austria) using custom scripts in RStudio. The R script and data set are available at the Open Science Framework repository (https://osf.io/6b4wq). Data are presented as the mean ± SD, and the significance level was set at α < 0.05.

Repeated measures within participants were modelled using a linear mixed‐effects model with random intercepts for participant identity. Fixed effects included energy system (aerobic, anaerobic alactic and anaerobic lactic), exercise protocol (CS and TS) and their interaction. Model residuals were checked using simulation‐based diagnostics, and no violations of assumptions were detected. The significance of fixed effects was assessed via type III ANOVA with Satterthwaite's degrees of freedom approximation. *Post hoc* pairwise comparisons were conducted using estimated marginal means with Holm correction.

For VL and an increase in lactate, assumptions of normality were tested using the Shapiro–Wilk test. Variables meeting the normality assumption (VL) were analysed using Student's paired *t*‐tests, with effect sizes reported as Hedges’ *g*. Variables violating normality (lactate) were analysed using Wilcoxon signed‐rank tests; for comparability, effect sizes were also expressed as Hedges’ *g*.

## 3 RESULTS

**FIGURE 2:**
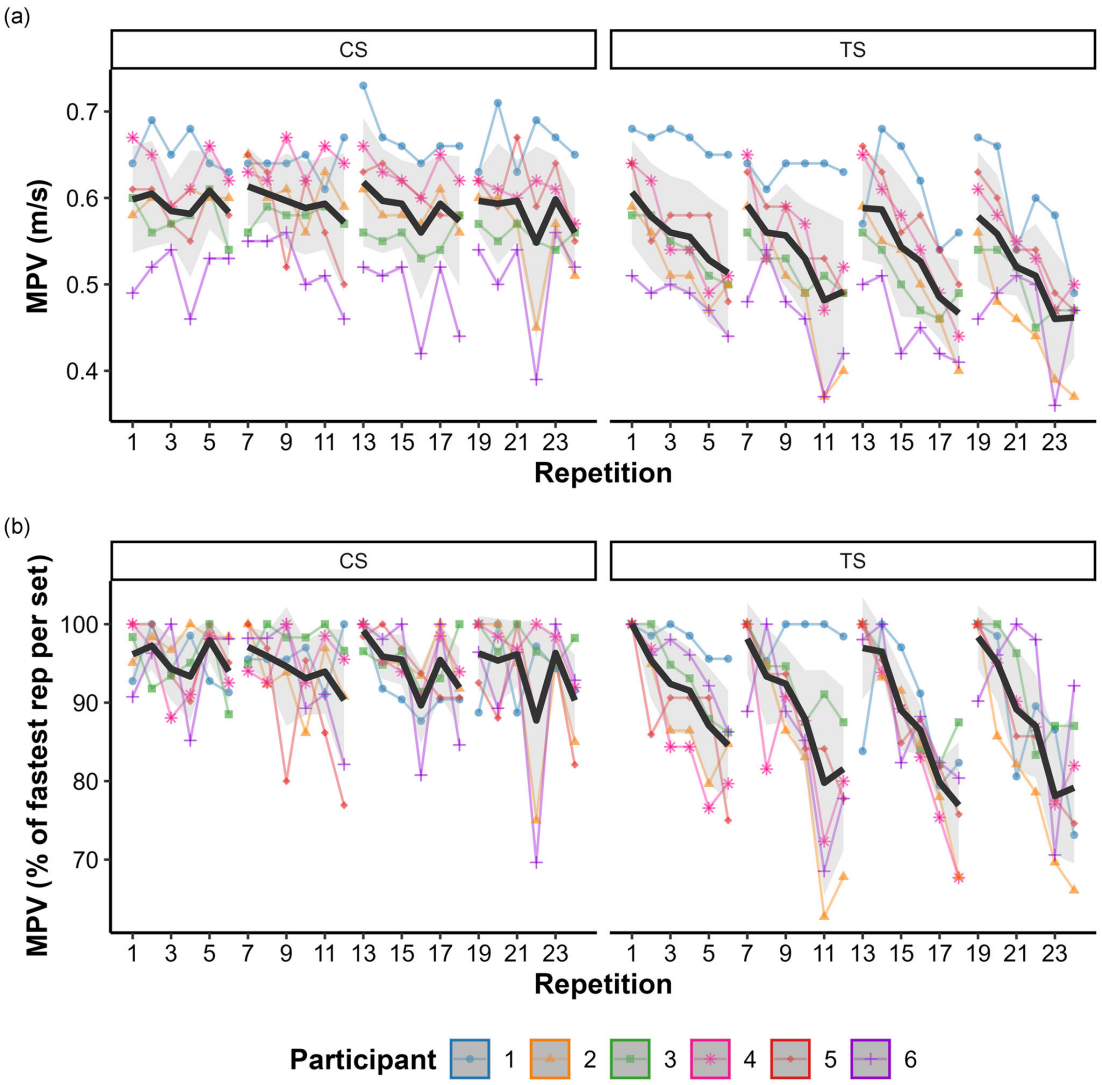
Mean propulsive velocity (MPV) across repetitions during the cluster set (CS) and traditional set (TS) protocols. The MPV is expressed as absolute values (a) and as a percentage of the fastest repetition within each set (b). Individual participant data are shown with thin coloured lines and symbols, with each colour representing one participant (*n* = 6). Black lines represent the mean values, and grey shaded areas indicate the SD. Student's paired *t*‐test revealed significant differences in velocity loss (*P* = 0.00275). Relative MPV values do not necessarily start at 100%, because the fastest repetition might occur at any position within a set; consequently, the mean value is not necessarily 100%.

The CS and TS protocols elicited distinct mechanical and metabolic responses. Mechanically, VL was lower during CS compared with TS (8.4% ± 3.4% vs. 19.4% ± 6.6%; Figure 2). Student's paired *t*‐test confirmed a significant difference between protocols [*t*(5) = −4.67, *P* = 0.003, *g* = −1.60], indicating a large effect.

**FIGURE 3:**
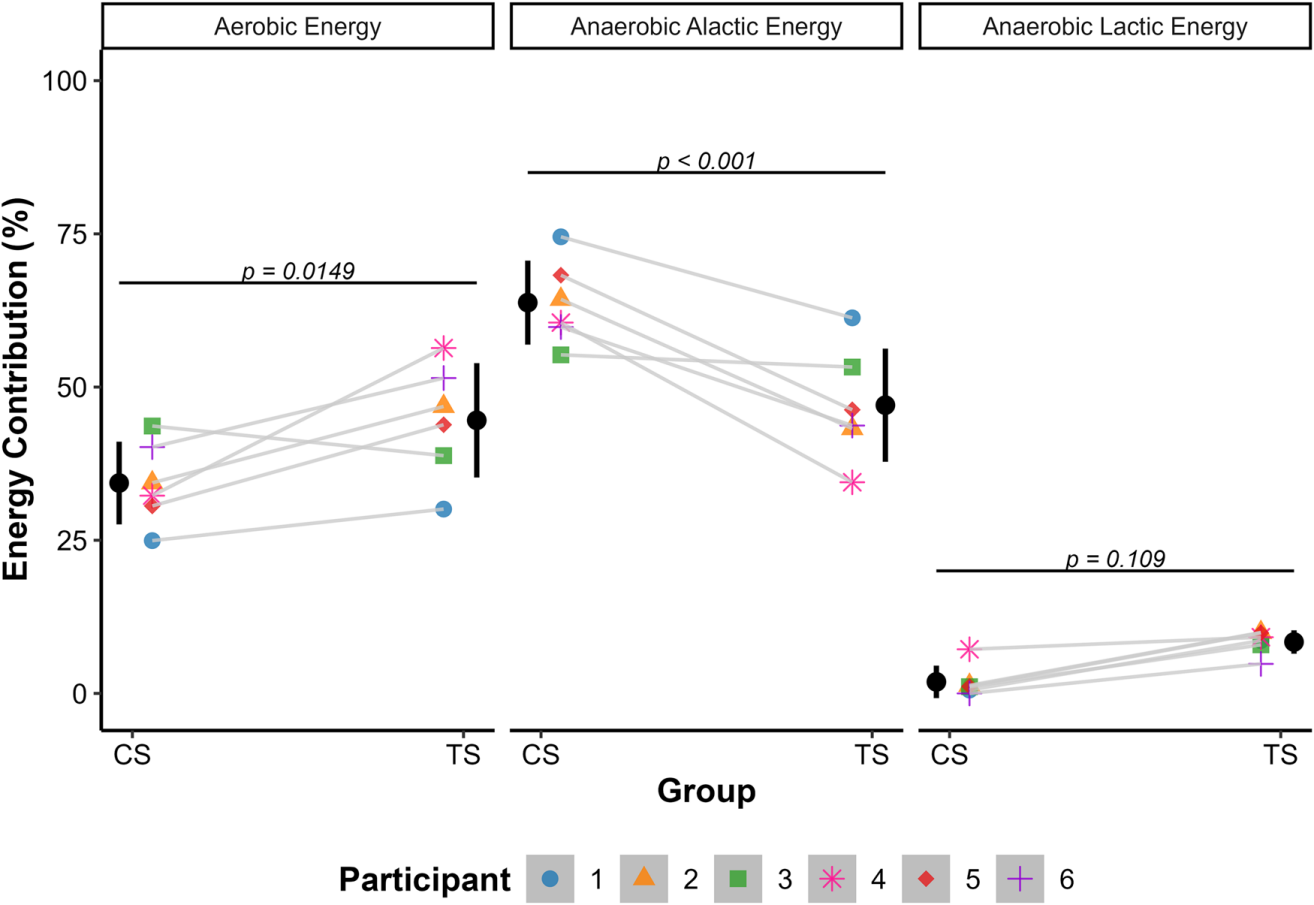
Individual comparison of relative energy contribution between cluster set (CS) and traditional set (TS) structures for the different energy systems (*n* = 6). Black vertical point ranges represent the mean ± SD. Individual participant data are shown as points connected by thin lines. Linear mixed‐effects modelling revealed an interaction between energy system and protocol (*P* < 0.001). Holm *post hoc* comparisons indicated significant differences for the aerobic contribution (*P* = 0.0149) and anaerobic alactic contribution (*P* < 0.001), whereas the anaerobic lactic contribution did not differ significantly (*P* = 0.109).

Metabolically, model‐based indices of energy system engagement differed between protocols. CS was characterized by a higher relative anaerobic alactic component (63.8% ± 6.9% vs. 47.0% ± 9.2%), whereas TS showed higher relative aerobic (44.6% ± 9.3% vs. 34.3% ± 6.7%) and anaerobic lactic components (8.4% ± 1.9% vs. 1.9% ± 2.6%). The linear mixed‐effects model revealed a significant interaction between energy system and exercise protocol [*F*(2,30) = 13.95, *P* < 0.001]. *Post hoc* comparisons indicated a significantly higher aerobic contribution for TS (*P* = 0.015) and a significantly higher anaerobic alactic contribution for CS (*P* < 0.001). The difference in the anaerobic lactic component did not reach statistical significance (*P* = 0.109). As shown in Figure 3, individual response patterns were generally consistent across participants, with most individuals showing a higher anaerobic alactic component during CS and a higher relative anaerobic lactic component during TS, although some inter‐individual variability was observed.

The increase in blood lactate from rest to peak was significantly greater in TS compared with CS (3.38 ± 0.95 vs. 1.09 ± 1.48 mmol L^−1^; *P* = 0.031), with a large effect (*g* = 1.12).

## 4 DISCUSSION

As hypothesized, the CS structure resulted in lower VL and was associated with a greater reliance on the anaerobic alactic energy pathway in comparison to the TS structure. Importantly, the present findings should be interpreted within the assumptions of the PCr‐La‐O_2_ method, which provides model‐based indices of relative energy system engagement rather than direct measurements of ATP turnover or PCr utilization. Within this modelling framework, CS was characterized by a distinct acute mechanical–metabolic profile, marked by improved velocity preservation and lower peak blood lactate accumulation. These findings align with prior studies showing that extended rest can attenuate metabolic fatigue (García‐Ramos et al., 2020; Mora‐Custodio et al., 2018) and support the foundational rationale for CS training (Haff et al., 2008; Jukic et al., 2020, 2022; Tufano et al., 2016).

The energetic profiles observed in this study offer qualitative insight into how different set structures are associated with different acute physiological responses. In the TS protocol, the higher increase in blood lactate coincided with a greater relative aerobic contribution. Although blood lactate cannot be interpreted as a direct measure of intramuscular glycolytic ATP production, elevated lactate concentrations are commonly associated with high glycolytic flux and might contribute to feedback mechanisms that downregulate key glycolytic enzymes, such as phosphofructokinase, thereby reducing glycolytic ATP turnover and increasing compensatory oxidative metabolism (Leite et al., 2007, 2011). Within this context, TS appears to be characterized by a metabolic profile marked by greater relative engagement of glycolytic and aerobic pathways across successive repetitions. In contrast, the inclusion of short intra‐set rest periods in CS was associated, within the PCr‐La‐O_2_ modelling framework, with a greater relative contribution of the anaerobic alactic component. These rest intervals are likely to allow partial restoration of PCr availability between repetition clusters, supporting a higher instantaneous rate of ATP resynthesis and, consequently, better preservation of movement velocity at the same external load.

The acute mechanical and metabolic responses observed in the present study are consistent with previous investigations reporting lower VL and reduced blood lactate accumulation for CS structures compared with TS structures (Jukic et al., 2020; Varela‐Olalla et al., 2020). Although these findings demonstrate that set configuration systematically alters acute metabolic demands, current meta‐analyses indicate largely comparable long‐term adaptations in strength and hypertrophy between CS and TS training, with some evidence suggesting greater improvements in explosive performance following CS and enhanced strength endurance following TS (Davies et al., 2021; Jukic et al., 2021).

A major strength of this study is the application of the PCr‐La‐O_2_ method to estimate relative energy pathway contributions non‐invasively during resistance exercise. However, the interpretation of these model‐based indices requires caution, and several methodological aspects warrant consideration. First, the Valsalva manoeuvre, common in resistance exercise, might disrupt the continuous breathing assumed by the model, potentially introducing variability. Second, there is no standardized approach for determining baseline oxygen consumption, which complicates comparisons across studies (Edel et al., 2024; Franchini et al., 2016; Latzel et al., 2018). In this study, baseline oxygen uptake was derived from the bi‐exponential model fit to enhance time efficiency, yielding a mean baseline of 5.34 ± 0.94 mL kg^−1^ min^−1^ (Figure 1). This value differs from the commonly assumed baseline of ∼4.5 mL kg^−1^ min^−1^, highlighting the sensitivity of metabolic calculations. Third, data smoothing, time‐frame selection and the time‐independent estimation of anaerobic lactic energy contribution might influence quantitative outcomes, particularly in intermittent and short‐duration exercise, such as resistance exercise. Notably, similar intermittent high‐intensity exercises have applied this modelling approach beyond steady‐state conditions (Edel et al., 2024; Kaufmann et al., 2022; Latzel et al., 2018; Ulupınar et al., 2021, 2023, 2024). Importantly, the PCr‐La‐O_2_ method has been proposed as the only currently available non‐invasive approach that, in principle, allows a conceptual distinction between anaerobic alactic and anaerobic lactic components (Ambaum & Hoppe, 2025). To address the present lack of shared data‐processing methodology, all data and analysis scripts have been made openly available. Although the PCr‐La‐O_2_ method represents a promising non‐invasive approach for studying resistance exercise, our findings reinforce the need for standardized analytical procedures and cautious quantitative interpretation, particularly in intermittent, non‐steady‐state exercise. This study illustrates both the potential and the substantial methodological challenges associated with its application in this context and aims to support critical evaluation and methodological refinement in future research.

The primary limitation of this study is its small sample size (*n* = 6). Therefore, all inferential statistics were framed as exploratory, and the findings should be interpreted accordingly. Despite this limitation, the use of a robust crossover design strengthens internal validity by reducing between‐participant variability. The consistently large effects observed for VL and blood lactate responses across participants suggest systematic differences between set structures. These effect size estimates might serve as useful reference values for future studies, which should aim to confirm these findings in larger and more diverse samples, including the examination of sex‐based differences.

## 5 CONCLUSION

This study demonstrates that CS and TS structure elicit distinct acute mechanical and metabolic responses during resistance exercise. Within the assumptions of the PCr‐La‐O_2_ method, CS was associated with lower VL and greater relative contribution of the anaerobic alactic pathway, whereas TS was characterized by greater blood lactate accumulation. From a practical perspective, CS and TS configurations can be viewed as complementary tools, with CS favouring the maintenance of movement velocity and mechanical output, and with TS imposing a greater acute metabolic challenge. These acute differences might help to explain previously reported similarities and differences in training adaptations.

## AUTHOR CONTRIBUTIONS

The experiments were performed in the laboratory of the Department of Training and Exercise Science, Faculty of Sport Science, Ruhr University Bochum, Germany. Erik Hobein, Antonia Edel, Alexander Ferrauti and Thimo Wiewelhove: conception and design of the study. Erik Hobein and Antonia Edel: acquisition of data. Erik Hobein: data analysis and visualization. Erik Hobein and Ivan Jukic: drafting of the manuscript. All authors: interpretation of results and critical revision of the manuscript. All authors approved the final version of the manuscript and agree to be accountable for all aspects of the work in ensuring that questions related to the accuracy or integrity of any part of the work are appropriately investigated and resolved. All persons designated as authors qualify for authorship, and all those who qualify for authorship are listed.

## CONFLICT OF INTEREST

None declared.

## FUNDING INFORMATION

None.

## Data Availability

The dataset and analyses code are available at the Open Science Framework (https://osf.io/6b4wq).
